# Nanoencapsulation of Acetamiprid by Sodium Alginate and Polyethylene Glycol Enhanced Its Insecticidal Efficiency

**DOI:** 10.3390/nano12172971

**Published:** 2022-08-27

**Authors:** Asgar Ebadollahi, Bita Valizadeh, Saleh Panahandeh, Hadiseh Mirhosseini, Maryam Zolfaghari, Tanasak Changbunjong

**Affiliations:** 1 Department of Plant Sciences, Moghan College of Agriculture and Natural Resources, University of Mohaghegh Ardabili, Ardabil 5697194781, Iran; 2 Department of Plant Protection, Faculty of Agriculture, Shahid Bahonar University of Kerman, Kerman 7616913439, Iran; 3 Department of Chemistry, Faculty of Science, Shahid Bahonar University of Kerman, Kerman 7616913439, Iran; 4 Department of Plant Protection, Faculty of Agricultural Sciences, University of Guilan, Rasht 416351314, Iran; 5 Department of Pre-Clinic and Applied Animal Science, Faculty of Veterinary Science, Mahidol University, Nakhon Pathom 73170, Thailand

**Keywords:** acetamiprid, nanoencapsulation, sodium alginate, polyethylene glycol, toxicity

## Abstract

Nanoformulation has been considered one of the newly applied methods in integrated pest management strategies. In this research, a conventional neonicotinoid insecticide acetamiprid was nanoencapsulated via AL (Sodium Alginate) and PEG (Polyethylene Glycol) and tested against the elm leaf beetle *Xanthogaleruca luteola*. The synthesized particles had spherical-like morphology and nanoscale based on TEM (Transmission Electron Microscopy) and DLS (Dynamic Light Scattering). The encapsulation efficiency and loading percentages of acetamiprid in AL and PEG were 92.58% and 90.15%, and 88.46% and 86.79%, respectively. Leaf discs treated with different formulations by the leaf-dipping method were used for oral toxicity assays. The LC_50_ values (Lethal Concentration to kill 50% of insect population) of acetamiprid and Al- and PEG-nanoencapsulated formulations on third-instar larvae were 0.68, 0.04, and 0.08 ppm, respectively. Based on the highest relative potency, AL-encapsulated acetamiprid had the most toxicity. The content of energy reserve protein, glucose, and triglyceride and the activity of detoxifying enzymes esterase and glutathione S-transferase of the larvae treated by LC_50_ values of nanoformulations were also decreased. According to the current findings, the nanoencapsulation of acetamiprid by Al and PEG can increase its insecticidal performance in terms of lethal and sublethal toxicity.

## 1. Introduction

The elm leaf beetle, *Xanthogaleruca luteola* Müller (Coleoptera: Chrysomelidae), is one of the damaging insect pests of elm (*Ulmus* spp.) in central and southern Europe, North Africa, west and central Asia, southern Australia, and temperate areas in North and South America [1]. Both the larvae and adults of *X. luteola* feed on the emergent leaves, and repeated infestations make the tree susceptible to different pests and diseases [2]. Although the use of synthetic chemicals is a primary method in the management of such insect pests, their excessive utilization can result in serious side-effects, including the spread of toxic ingredients in the environment and threats to non-target organisms [3,4,5]. Accordingly, the introduction of novel and efficient formulations to decrease the number of active ingredients and their detrimental side-effects is necessary for insect pest management strategies [6]. The pesticide encapsulation based on the controlled-release technique was considered an effective way to improve their stability [7,8]. Furthermore, encapsulated formulations are expected to diminish the active ingredients’ utilization compared to non-capsulated materials [9].

An encapsulation process based on nanotechnology has extensive applications in industries from foods to cosmetics, paper, paints, agriculture, etc. [6,10,11,12]. Encapsulation of the active ingredients with other materials can envelope sensitive ingredients into a matrix to protect from adverse factors such as air or light [13]. Nanoencapsulation has also been considered for applicable formulations of pesticides in recent years [14,15], in which several techniques such as chemical vapor deposition, electrochemical, microwave-assisted and sol–gel synthesis, and ultra-sonication were used for nanoparticles’ synthesis [16].

Sodium alginate (AL) is a linear natural bio-polysaccharide extracted from brown algae and has good water solubility, biocompatibility, and low toxicity [17]. It has been reported that AL can improve the solubility of hydrophobic ingredients [18,19] and inhibit the photo-degradation of active compounds [20,21]. Based on the low cost, availability, and biodegradability, synthetic polymer polyethylene glycol (PEG) has been recommended for nanoencapsulation of pesticides [22]. PEG has several remarkable properties, including biocompatibility, biodegradability, and solubility in organic solvents and water [23]. Indeed, the promising potential of PEG-based micro/nanoparticles for agricultural, food, and medical applications was reported [24,25,26]. Thus, AL and PEG were chosen as hydrophilic pesticide carriers in this study.

Acetamiprid is an odorless neonicotinoid insecticide with a chloropyridinyl group that acts on the acetylcholine (nACh) receptors, which are used for pest control on fruit and unfruitful trees, leafy vegetables, and ornamental plants [27,28]. It is an organic compound with 222.67 g/mol molar mass and the chemical formula C_10_H_11_ClN_4_. This neonicotinoid insecticide is usually a xenobiotic and can have detrimental impacts on non-target insect predators and vertebrates [29,30,31]. Accordingly, acetamiprid, effective on several insect pests, has harmful side-effects and should be sustainably used with low concentration. 

Nanoencapsulation of pesticides emerges as a developing field in pest management strategies by low concentration of active agents, making them less toxic to non-target organisms along with long-term availability to the target pests [8,32]. Therefore, the present study was conducted to achieve the successful nanoencapsulation of acetamiprid based on AL and PEG for control of elm leaf beetle by application of low active-ingredient concentration and more sustainable formulations.

## 2. Materials and Methods

### 2.1. Insect Rearing

Eggs and larvae of the elm leaf beetle, *X. luteola*, were collected from elm trees (*Ulmus americana* L.) in the city park of Rasht, northern Iran. The larvae were reared in plastic boxes (10–20 cm) in a rearing chamber set at 25 ± 2 °C, 14–10 h light–dark schedule, and 65% relative humidity. Fresh elm leaves were provided daily for feeding. Adults were reared similarly, and their eggs were used to maintain the culture. Newly emerged 3rd-instar larvae (<24 h) were used for bioassays.

### 2.2. Chemicals

Calcium chloride, polyvinyl alcohol (PVA), AL, PEG, d-mannitol, dioctyl sodium sulphosuccinate, and 1-chloro-2, 4-dinitrobenzene (CDNB) were purchased from Tebshahr Company, Tehran, Iran. Acetamiprid (20% SP: ‘A soluble powder formulation containing 20% of the active ingredient’) was taken from Aroxa Crop Science Private Limited, Dhamatwan, India.

### 2.3. Preparation of AL–Acetamiprid Nanoparticles

Nanoparticles were synthesized by emulsion cross-linking technology, according to Chavanpatil et al. [33]. A 10 mL measure of sodium alginate solution in water (0.1:1.0% *w/v*) was emulsified in dioctyl sodium sulfosuccinate dissolved in 30 mL methylene chloride (0.05:20% *w/v*) through sonication above the ice bath for 1 min. The emulsion was emulsified into 150 mL of aqueous PVA solution (5% *w/v*) by sonication above the ice bath for 1 min to form a secondary water-in-oil-in-water emulsion. The emulsion was stirred using a magnetic stirrer, and 50 mL of aqueous calcium chloride solution (60% *w/v*) was then added and additionally stirred for 18 h. To evaporate methylene chloride, the emulsion was stirred under a vacuum for 1 h. To prepare insecticide sodium alginate nanoparticles, 150 mg acetamiprid was dissolved in the aqueous alginate solution by sonication. The formed nanoparticles were isolated by ultracentrifugation at 15,000 rpm, washed twice with deionized water to eliminate PVA and unentrapped acetamiprid, and then re-suspended in water. Finally, the protectant d-mannitol (5%, *w/v*) was added and the particles lyophilized at 0.0010 mbar pressure and −90 °C for 24 h.

### 2.4. Preparation of PEG–Acetamiprid Nanoparticles

Briefly, 0.2 g acetamiprid was dissolved in 12 mL PEG. Hydroxypropyl methylcellulose solution was prepared by adding 0.2 g in 20 mL of distilled water to the acetamiprid-PEG solution. To achieve a homogeneous solution, the final solution was stirred for 2 h at 300 rpm under room temperature.

### 2.5. Description of Nanoparticles

The size and morphology of AL– and PEG–acetamiprid nanoparticles were assessed by Transmission Electron Microscopy (TEM; Zeiss Leo 906 TEM-Gmbh, Oberkochen, Germany) based on the negative-staining method [23]: TEM analysis was acquired at an accelerating voltage of 120 kV. For the sample preparation procedure, 1 mg of the sample was dispersed in 1 mL H_2_O, and then one drop of the nanoparticle suspension was poured on a carbon-coated copper grid. Molecular aggregates were permitted to resolve on the grid for 3 min. The surplus liquid was blotted with a filter paper strip. Afterward, a drop of phosphotungstic acid (2%) was poured to the grid. The extra stain was removed after 1 min, and the grid was dried at room temperature. The particles were also specified thrice by Dynamic Light Scattering (DLS) using a spectroscopy instrument (Zetasizer Nano-ZS90 system, Malvern, Worcestershire, UK). For data analysis and data collection, the size distribution data were readily acquired from the software of the DLS instrument. The analysis of the DLS data were performed by cumulant method, which enables the determination of the average particle size in a nanoparticle sample. FTIR analysis (100 Spectrum, Perkin Elmer, Rodgau, Germany) was carried out by the KBr pellet method and the presence of the various vibrational modes in the synthesized nanoparticles was investigated [34].

### 2.6. Oral Toxicity Assay

Oral toxicity was assessed on newly emerged 3rd-instar larvae of *X. luteola* with different concentrations of acetamiprid (0.25, 0.50, 0.75, 1.50, and 4.00 ppm) and AL– (0.01, 0.03, 0.06, 0.12, and 0.18 ppm) and PEG–acetamiprid nanoparticles (0.01, 0.05, 0.10, 0.25, and 0.50 ppm), which were selected based on preliminary experiments. The fresh elm leaf discs (4 cm × 7 cm) were dipped in desired concentrations for 30 s and dried at room temperature for 30 min. Treated leaf discs were positioned in 9 cm Petri dishes, and larvae were transferred to feed for 24 h. In each experiment, ten larvae were tested in 3 replicates. Water-dipped leaf discs were used as control, and the mortality was recorded after 24 h.

### 2.7. Preparation of the Whole-Body Homogenates for Biochemical Analysis

Third-instar larvae treated by nanoencapsulated acetamiprid with LC_30_ and LC_50_ values were killed by freezing after 24 h of the treatment. The whole body was homogenized in 1 mL of universal buffer and was centrifuged for 10 min at 13,000× *g*. The supernatant was transferred to new tubes and stored at −20 °C until used. Each biochemical analysis was repeated three times.

### 2.8. Energy Reserves

The amount of whole-body protein in the alive insects treated by LC_30_ and LC_50_ of all formulations was assessed by the method of Bradford [35]. In this technique, proteins made a compound purplish blue with an alkaline copper solution, which, with its absorption value at 540 nm, is related to the amount of the whole-body protein. The amount of glucose in treated larvae was examined according to the techniques of Siegert [36] by automated enzymatic analyses using a glucose assay kit (Biochem Co., Tehran, Iran). Reagents A and B were mixed (4:1), followed by addition of samples. The absorbance was read at 492 nm in an ELISA reader (Awareness, Temecula, CA, USA). The amount of triglyceride was estimated according to the method of Fossati and Prencipe [37] with a triglyceride kit (Biochem Co., Tehran, Iran). The reducing absorption rate for triglyceride analysis was read at 545 nm.

### 2.9. Detoxifying Enzymes

Glutathione S-Transferase (GST) and general esterases as key detoxifying enzymes in insect pests were estimated according to the methods of Van Asperen [38] and Oppenorth et al. [39], respectively. For determination of the glutathione S-transferase (GST) activity, 1-chloro-2, 4-dinitrobenzene (CDNB) (20 mM) was used as the substrate. A 15 µL measure of the supernatant was mixed with 135 µL of phosphate buffer (pH 7) and 50 µL of CDNB. The absorbance was read at 340 nm. For general esterases, Alpha-naphtylacetate (α-NA) and β-naphtylacetate (β-NA) (10 mM) were used as substrates. One gut from each treated insect was homogenized with 1000 µL 0.1 M phosphate buffer (pH 7) containing 0.01% Triton x-100. This solution reacted with the substrate, and by using a dye indicator (Fast Blue RR salt) (1 mM) a colored solution was formed and the absorbance was read at 630 nm.

### 2.10. Data Analysis

The normality of mortality data was tested by Kolmogorov–Smirnov and data were then subjected to analysis of variance. Probit analyses to calculate lethal concentrations and regression-line details were used for all formulations by Polo-Plus (LeOra Software, Berkeley, CA, USA). Relative potency (RP) for each mixture was determined on the basis of acetamiprid (lowest toxicity) and calculated using the following formula: RP = LC_50_ of acetamiprid/LC_50_ of individual other compounds. Raw data obtained from the biochemical analysis were subjected to a one-way statistical analysis of variance test for significant differences in the measured parameters. The Tukey–Kramer test at a 5% significance level was used to compare means using SAS statistical software (SAS Institute, Cary, NC, USA). Encapsulation efficiency and loading percentages were evaluated according to the following formula [40]:

$$\mathrm{Efficiency}\;\mathrm{Percentage}\;\mathrm{Encapsulation}=\frac{\mathrm{weight}\;\mathrm{of}\;\mathrm{encapsulated}\;\mathrm{acetamiprid}}{\mathrm{weight}\;\mathrm{of}\;\mathrm{acetamiprid}\;\mathrm{used}\;\mathrm{initially}}\times 100$$

$$\mathrm{Loading}\;\mathrm{Percentage}\;=\frac{\mathrm{weight}\;\mathrm{of}\;\mathrm{encapsulated}\;\mathrm{acetamiprid}}{\mathrm{weight}\;\mathrm{of}\;\mathrm{acetamiprid}-\mathrm{weight}\;\mathrm{of}\;\mathrm{encapsulated}\;\mathrm{particles}}\times 100$$

## 3. Results

### 3.1. Characterization of Synthesized Nanoparticles

**Figure 1:**
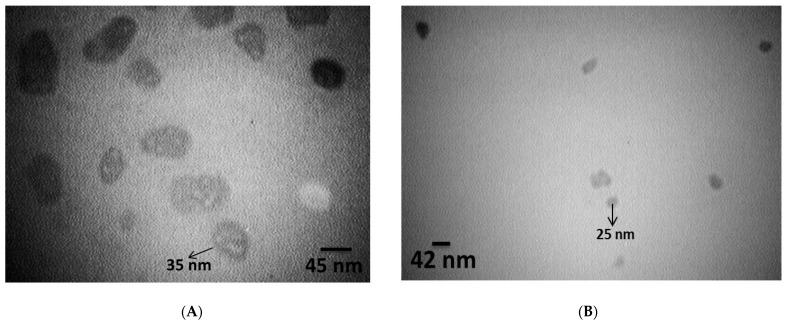
TEM images of AL–acetamiprid (**A**) and PEG–acetamiprid (**B**) nanoparticles.

**Figure 2:**
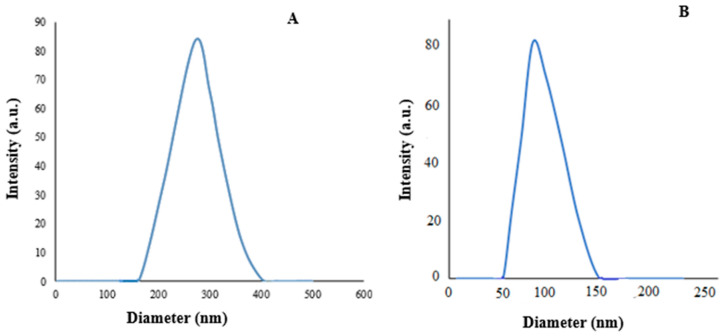
DLS measurement of particle size distribution of AL–acetamiprid (**A**) and PEG–acetamiprid (**B**) nanoparticles.

The surface morphology and size of AL– and PEG–acetamiprid were investigated by TEM, in which elliptical shapes of AL– and PEG–acetamiprid were distinguished. Although there were various particle sizes, an approximate size of 46.13 and 25.66 nm could be detected for AL– and PEG–acetamiprid nanoparticles, respectively (Figure 1). DLS analysis was also used to measure the particle sizes of AL– and PEG–acetamiprid through the colloidal solutions. As shown in Figure 2, a z-average particle size of 270.5 and 101.2 nm with a polydispersity index (PDI) of 0.462 and 0.350 were obtained for AL- and PEG-based nanoparticles, respectively. PDI values less than <0.5 are good for detecting the size distribution of colloidal suspension and suitable for the DLS technique [41], which was obtained in this study. DLS sizes are larger than those obtained by TEM for corresponding nanoparticles because the nanoparticles are solvated in the solution phase in the DLS analysis. Therefore, the nanoparticles may be aggregated in the colloidal solution.

**Figure 3:**
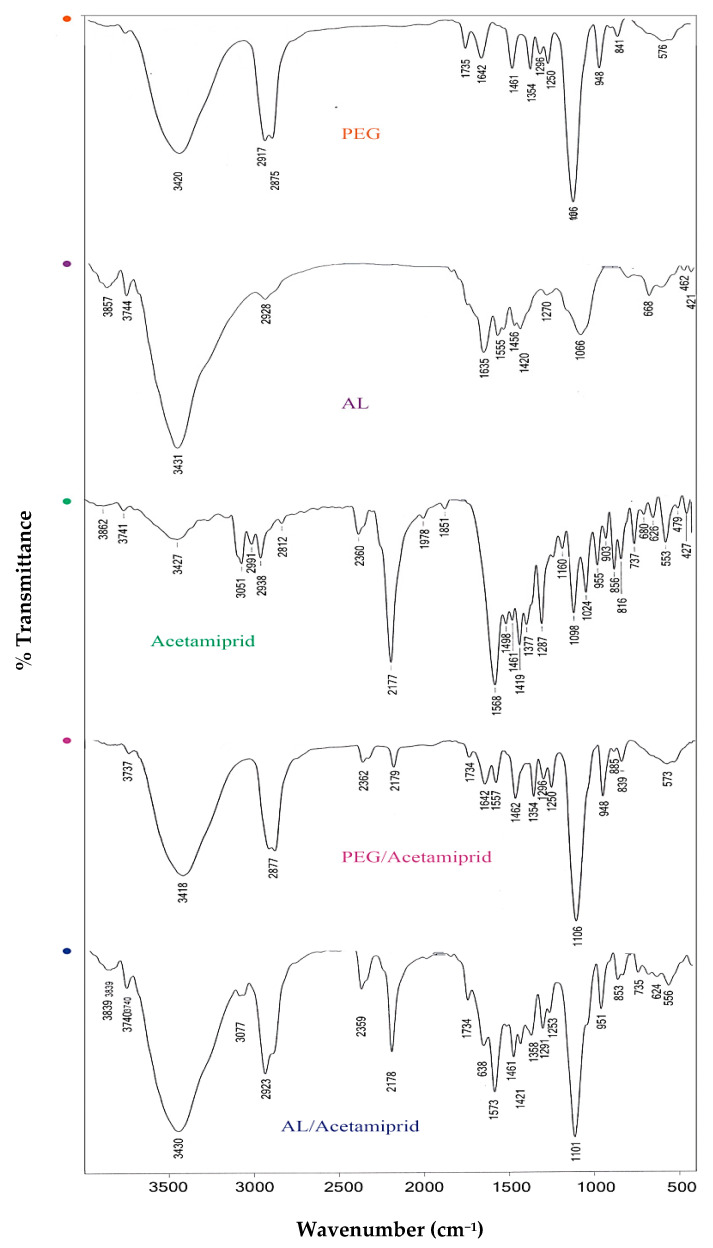
FTIR spectra of PEG, AL, Acetamiprid, AL–acetamiprid, and PEG–acetamiprid.

FTIR spectroscopy was applied to confirm the formation of synthesized nanoparticles. Therefore, the functional groups of AL, PEG, acetamiprid, and AL– and PEG–acetamiprid nanoparticles were identified using FTIR spectra (Figure 3). The AL-based nanoparticles showed frequency bands at about 1420 and 1635 cm^−1^, which are assigned to symmetric and asymmetric stretching vibrations of carboxylate groups. The presence of a peak at a wavenumber of 2923 cm^−1^ corresponds to the asymmetric aliphatic CH stretching bands. The adsorption peak in the range of 3000–3600 cm^−1^ in the spectrum of AL is attributed to the OH stretching vibration. Moreover, the frequency peaks at 1066 cm^−1^ correspond to the C–O bond stretching vibration of the pyranose ring. For acetamiprid, the characteristic peaks at 2177 and 1568 cm^−1^ are related to –CN and C=N groups stretching vibrations, respectively. The C–H bond stretching vibrations were observed in the range of 2600–3000 cm^−1^. The adsorption peak at around 1098 cm^−1^ is related to the C–Cl bond stretching. In addition, the broad peak at 3300–3500, and 1377 cm^−1^ are ascribed to NH and C–N stretching vibration, respectively. According to the spectrum of PEG, the bands located at 1461, 1250, and 1106 cm^−1^ are assigned to the C–H bending vibration, C–O–C vibrational elongation, and stretching vibration of the C–O bond, respectively. Additionally, the peaks at 2875 and 2917 cm^−1^ are ascribed to the C–H stretching vibrations. Furthermore, the adsorption band at 3420 cm^−1^ is related to the stretching vibration of a hydroxyl group. The FTIR spectrum of AL– and PEG–acetamiprid nanoparticles showed some characteristic absorption bands of AL and PEG, respectively. For example, in the AL–acetamiprid spectrum, the characteristic absorption bands of AL, including symmetric and asymmetric stretching vibrations of carboxylate groups at 1420 cm^−1^ and 1635 cm^−1^, existed. Furthermore, in the spectrum of PEG-acetamiprid, the bands in the wavenumbers of 2875 and 2917 cm^−1^ are related to C–H stretching vibrations of PEG. However, it is seen that some of the absorption peaks of AL and PEG were not observed in the spectrum of AL– and PEG–acetamiprid due to overlapping their peaks with acetamiprid peaks. The results of the FTIR analysis confirmed the successful loading of AL and PEG into the acetamiprid.

The encapsulation efficiency percentage (EE%) and loading percentage of acetamiprid in AL and PEG were 92.58% and 90.15%, and 88.46% and 86.79%, respectively.

### 3.2. Larvicidal Activity

Based on the results of a Kolmogorov–Smirnov test, the mortality of *X*. *luteola* larvae treated by acetamiprid (Z = 0.642: two-tailed Significant = 0.804), Al–acetamiprid (Z = 0.595: two-tailed Significant = 0.871), and PEG–acetamiprid (Z = 0.585: two-tailed Significant = 0.883) had statistically normal distributions. Analysis of variance was also showed that tested concentrations of acetamiprid (F = 19.00), AL–acetamiprid (F = 24.38), and PEG–acetamiprid (F = 36.57) had significant effects on the larval mortality (df = 4, 14: *p* < 0.001). 

The LC_50_ values of acetamiprid, Al–acetamiprid, and PEG–acetamiprid were 0.68, 0.04, and 0.08 ppm, respectively. It can be assumed that the toxicity of acetamiprid was augmented after nanoencapsulation by Al and PEG. Indeed, based on the lowest LC50 value and the highest relative potency, AL–acetamiprid was more toxic than acetamiprid and PEG–acetamiprid against third-instar larvae of *X*. *luteola* (Table 1).

### 3.3. Energy Reserves

The effect of AL– and PEG–acetamiprid nanoparticles and the acetamiprid on the energy reserves of *X. luteola* larvae are presented in Table 2. Increasing concentration of AL- and PEG-based nanoparticles caused a significant reduction in the content of all energy reserves: protein, glucose, and triglycerides. For instance, by increasing the AL–acetamiprid concentration from LC_30_ to LC_50_, the glucose content was reduced to 33%, and a 1.1-fold increase in PEG–acetamiprid concentration caused a 28% decrease in glucose levels (Table 2).

### 3.4. Detoxifying Enzymes

The effects of acetamiprid and AL– and PEG–cetamiprid nanoparticles on the esterase and GST activity of *X. luteola* are shown in Table 3. Both detoxifying enzymes’ content was significantly decreased by using LC_50_ values of nanoparticles in comparison with the control groups (Table 3).

## 4. Discussion

Nanoencapsulation based on the controlled-release technique has the potential to provide stable formulations that are more efficient [8]. The main objective of active material nanoencapsulation is to maintain its properties and avoid deterioration [42]. The improvement of toxicity effects and stability of insecticides by nanoencapsulated formulations has been reported in recent studies. For example, nanoencapsulation of acetamiprid by porous silica nanoparticles (Ace@MSNs) increases its insecticidal efficiency and decreases the pesticide residue: testing LC_50_ value of nanoencapsulated acetamiprid against tea aphids was three times lower than that of the commercial preparation, and the average retained concentrations in tea leaves treated by acetamiprid were about 1.3 times those in the nanoformulation [28]. In this study, it was found that the nanoencapsulation of acetamiprid using coating materials AL and PEG enhanced the lethal and sublethal efficiency of insecticide acetamiprid. The nanoparticles were prepared based on natural materials AL and PEG, which were approved for agricultural practice and have eco-friendly features [43,44,45]. Adak et al. [46] indicated that PEG-based nanoformulations of neonicotinoid insecticide imidacloprid can be used for efficient pest management according to their high solubilization power and low critical micelle concentration. Although the encapsulation efficiency of PEG-based nanoformulations (60.0–97.9%) reported by Adak et al. [47] are in accordance with present findings (90.1%), the loading capacity (6.8–60.0%) was lower than our finding (86.8%). In the other study, 95% encapsulation efficiency and 78% loading capacity of a pyrethroid insecticide cypermethrin in AL-based nanoformulation were reported [47], which was approximately in agreement with our findings (92.58% and 88.46%, respectively). Mentioned differences may be due to different chemical compositions of tested insecticides and their encapsulation methods. Furthermore, although the nanoscale size measured in this study is consistent with data provided for nanoencapsulation of other insecticides by the same polymers [46,47], changes in encapsulation methods can give different-sized particles.

The nanoscale of synthesized AL– and PEG–acetamiprid in the present study, attained by both TEM and DLS, were confirmed by those reported for the AL- and PEG-encapsulated temephos and imidacloprid [48]. The nanoscale of the PEG-encapsulated acetamiprid in the present study is also in good agreement with PEG-based encapsulated cinnamon essential oil [49]. The expansion in the average diameter achieved by DLS may be qualified by nanoparticle aggregation compared to the TEM results. According to DLS, the average hydrodynamic diameter of AL–acetamiprid nanoparticles was larger than the PEG–acetamiprid nanoparticle, which may be attributed to the solubility of acetamiprid in an aqueous system [50]. Furthermore, characteristics of the synthesized AL– and PEG–acetamiprid nanoparticles were also described by FTIR, in which the presence of the insecticide in the nanoformulation was confirmed.

In this investigation, the nanoencapsulated formulations based on AL and PEG showed higher toxicity against larvae of *X. luteola* after 24 h than acetamiprid. Takei et al. [51] indicated that the polylactide microsphere is a promising capsulation agent for acetamiprid, in which the pesticide release from microspheres was improved by adding poly-є-caprolactone, but it has not been checked against insect pests or any other damaging agents. However, similar to the present findings, the performance of nano-acetamiprid synthesized based on poly-є-caprolactone against the pathogenic fungi *Aspergillus niger* was many-fold times more effective than the typical commercial acetamiprid [30].

The enzymatic activity and energy reserves in the third-instar larvae of *X. luteola* feed on elm leaf leaves treated by AL– and PEG–acetamiprid nanoparticles were reduced in this study. Key roles of protein in insect digestion and energy conversion and metabolism have been found [52,53]. For example, it was indicated that a reduction in protein content in insect larvae treated with bio-pesticides could lead to decreased growth hormone levels [54]. In the present study, the protein content of *X. luteola* larvae was significantly decreased by free and nanoencapsulated acetamiprid, in which PEG-based nanoencapsulated insecticide was more efficient than others. Therefore, using such nanoencapsulated insecticides can cause significant disruption in the function of proteins. As lipids provide reserve energy after feeding and play a crucial role in intermediary metabolism, they are essential macromolecules in insect physiology [55]. The triglyceride content of *X. luteola* larvae was also reduced by both free and nanocapsulated formulations of acetamiprid, which may be caused by variations in synthesis patterns along with hormonal dysfunction in its metabolism [13]. Glucose, the key monosaccharide in insects, was also significantly reduced in *X. luteola* larvae treated with AL– and PEG–acetamiprid nanoparticles. A decrease in glucose content may be attributed to low feeding in treated larvae [56]. In general, the reduction in protein, lipid, and glucose resources of insect pest larvae may affect survival and reproduction parameters, including egg production, fecundity, and fertility, even in later generations [57,58]. Esterases and glutathione S-transferases are detoxifying enzymes that are involved in the falling of exogenous agents’ impacts [59]. The activity of these detoxifying enzymes was decreased in larvae treated by both free and nanocapsulated acetamiprid in the present study. It was also found that the activity of esterases and glutathione S-transferases was more affected by AL– and PEG–acetamiprid nanoparticles than by the pure-insecticide formulation. The low activity of these enzymes could be related to the interruption of their production, causing more susceptibility to insect pests [60].

Generally, nanoencapsulated formulations can decrease the required concentration of insecticide, resulting in low human threat and environmental pollution and a reduction in the cost of plant protection strategies. In this study, a nanoencapsulation technique was developed to effectively produce AL- and PEG-nanoparticles. These nanoparticles are low-cost and eco-friendly carriers in the controlled release of acetamiprid insecticide. Although AL and PEG are non-toxic and safe for agricultural practice, their safety typically increases by formulating microscopic particles [61]. Our findings displayed that the AL- and PEG-based encapsulated acetamiprid has a higher toxicity than pure formulation against *X. luteola* larvae. It can also be concluded that AL– and PEG–acetamiprid nanoparticles with feasible application have high insecticidal effectiveness against *X. luteola* at much lower concentrations than those required for non-encapsulated formulation. The specific features of AL– and PEG–acetamiprid nanoparticles, including their ability to dissolve in water and the augmentation of active ingredient efficiency, distinguish their promising potential in the management of *X. luteola*. However, additional research is recommended to check the possibility of loading conventional insecticide and other active agents in AL and PEG and their toxicity to other insect pests. In addition, the slow release of insecticides can lead to an increase in the resistance of the pest population, which should be considered.

## Figures and Tables

**Table 1:** Probit analysis of free and nanoencapsulated acetamiprid based on AL and PEG on third-instar larvae of *Xanthogaleruca luteola*.

Bioassay	LC_30_ (95% Confidence Limits) (ppm)	LC_50_ (95% Confidence Limits) (ppm)	Slope ± SE	Intercept ± SE	χ² (df = 3)	Relative Potency
AL–acetamiprid	0.015 (0.005–0.028)	0.048 (0.025–0.076)	1.071 ± 0.198	−0.731 ± 0.211	2.734 *	14.16
PEG–acetamiprid	0.024 (0.008–0.048)	0.081 (0.046–0.135)	1.002 ± 0.196	−0.913 ± 0.219	0.321 *	8.39
Acetamiprid	0.338 (0.191–0.475)	0.680 (0.486–0.915)	1.729 ± 0.304	−3.170 ± 0.588	0.838 *	1.00

* According to Chi-square values, no heterogeneity factor was used in the calculation of confidence limits. The LC_30_ values were selected for sublethal bioassays. LC: lethal concentration (ppm), and df: degrees of freedom. Relative potency = LC_50_ of Acetamiprid/LC_50_ of other compounds.

**Table 2:** Effect of free and nanoencapsulated acetamiprid based on AL and PEG on macromolecules in third-instar larvae of *Xanthogaleruca luteola*.

Bio-Assay	Concentrations	Protein (mg/dL)	Glucose (mg/dL)	Triglyceride (mg/dL)
Acetamiprid	Control	1.233 ± 0.0360 ^a^	0.0933 ± 0.0047 ^a^	1.8900 ± 0.0145 ^a^
	LC_30_	1.0900 ± 0.0030 ^b^	0.0790 ± 0.0208 ^a^	1.633 ± 0.2185 ^ab^
	LC_50_	1.0566 ± 0.0098 ^b^	0.0733 ± 0.0317 ^b^	1.5100 ± 0.0965 ^b^
	F-Value	10.94	49.80	5.04
	Pr	0.0018	0.0001	0.0300
AL–acetamiprid	LC_30_	0.9700 ± 0.0057 ^b^	0.0623 ± 0.0090 ^a^	1.5557 ± 0.0431 ^a^
	LC_50_	0.9433 ± 0.0088 ^b^	0.0433 ± 0.0083 ^b^	1.1600 ± 0.0677 ^b^
	F-Value	2.19	29.51	17.65
	Pr	0.0170	0.0001	0.0005
PEG–acetamiprid	LC_30_	0.8500 ± 0.0012 ^c^	0.0690 ± 0.0008 ^a^	1.433 ± 0.1185 ^ab^
	LC_50_	0.8366 ± 0.0098 ^c^	0.0513 ± 0.0017 ^b^	1.3000 ± 0.0765 ^b^
	F-Value	12.84	46.10	5.10
	Pr	0.0019	0.0001	0.0300

In each separate column, the means with different superscript letters designate significant differences at *p* < 0.05 according to Tukey’s test.

**Table 3:** Effect of pure and nanoencapsulated acetamiprid on the activity of glutathione S-transferase (GST) and esterase in third-instar larvae of *Xanthogaleruca luteola*.

Bio-Assay	Concentrations	Esterase (U/mg Protein)	GST (U/mg Protein)
Acetamiprid	Control	0.02300 ± 0.001 ^a^	0.0853 ± 0.004 ^a^
	LC_30_	0.0065 ± 0.0015 ^b^	0.06366 ± 0.002 ^ab^
	LC_50_	0.0001 ± 0.00001 ^b^	0.05700 ± 0.001 ^b^
	F-Value	21.76	11.13
	Pr	0.0003	0.0483
AL–acetamiprid	LC_30_	0.0010 ± 0.0001 ^b^	0.0506 ± 0.0063 ^b^
	LC_50_	0.0001 ± 0.0000 ^c^	0.0490 ± 0.0024 ^b^
	F-Value	28.13	22.87
	Pr	0.0001	0.0005
PEG–acetamiprid	LC_30_	0.0018 ± 0.0001 ^b^	0.0696 ± 0.0053 ^ab^
	LC_50_	0.0003 ± 0.0000 ^c^	0.0580 ± 0.0021 ^b^
	F-Value	30.23	21.27
	Pr	0.0001	0.0005

In each separate column, the means with different superscript letters designate significant differences at *p* < 0.05 according to Tukey’s test.

## Data Availability

Not applicable.

## References

[1] Lefoe G., Dominiak B., Worsley P., Davies J. (2014). Elm Leaf Beetle “*Xanthogaleruca luteola*” (Muller) Dispersal across South Eastern Australia (1989–2011). Plant Prot. Q..

[2] Chiffelle Í., Huerta A., Bobadilla V., Macuada G., Araya J.E., Curkovic T., Ceballos R. (2019). Antifeedant and insecticidal effects of extracts from *Melia azedarach* fruits and *Peumus boldus* leaves on *Xanthogaleruca luteola* larvae. Chil. J. Agri. Res..

[3] Singh N.S., Sharma R., Parween T., Patanjali P.K., Oves M., Zain Khan M., Ismail I. (2018). Pesticide contamination and human health risk factor. Modern Age Environmental Problems and their Remediation.

[4] Tudi M., Ruan H.D., Wang L., Lyu J., Sadler R., Connell D., Chu C., Phung D. (2021). Agriculture Development, Pesticide Application and Its Impact on the Environment. Int. J. Environ. Res. Public Health.

[5] Alengebawy A., Abdelkhalek S.T., Qureshi S.R., Wang M.Q. (2021). Heavy metals and pesticides toxicity in agricultural soil and plants: Ecological risks and human health implications. Toxics.

[6] Natrajan D., Srinivasan S., Sundar K., Ravindran A. (2015). Formulation of essential oil-loaded chitosane alginate nanocapsules. J. Food Drug Anal..

[7] Liu J.W., Lu Y. (2006). Colorimetric sensing of adenosine and cocaine based on a general sensor design involving aptamers and nanoparticles. Angew. Chem. Int. Ed..

[8] Nuruzzaman M., Rahman M.M., Liu Y., Naidu R. (2016). Nanoencapsulation, nano-guard for pesticides: A new window for safe application. J. Agric. Food Chem..

[9] Sansukcharearnpon A., Wanichwecharungruang S., Leepipatpaiboon N., Kerdcharoen T., Arayachukeat S. (2010). High loading fragrance encapsulation based on a polymerblend: Preparation and release behavior. Int. J. Pharm..

[10] Liu S.Q., Yuan L., Yue X.L., Zhang Z.Z., Tang Z.Y. (2008). Recent Advances in nanosensors for organophosphate pesticide detection. Adv. Powder Technol..

[11] Wu W.Y., Bian Z.P., Wang W., Zhu J. (2010). Gold Nanoparticle composite film-based silver enhanced colorimetric detection of cardiac troponin. Sens Actuators B Chem..

[12] Horwat D.I., Zakharov J., Endrino L. (2011). Chemistry, Phase formation, and catalytic activity of thin palladium-containing oxide films synthesized by plasma-assisted physical vapor deposition. Surf. Coat..

[13] Valizadeh B., Samarfard S., Sendi J.J., Karbanowicz T.P. (2020). Developing an *Ephestia kuehniella* hemocyte cell line to assess the bio-insecticidal potential of microencapsulated *Helicoverpa armigera* Nucleopolyhedrovirus against cotton bollworm (Lepidoptera: Noctuidae) Larva. J. Eco. Entomol..

[14] Chaud M., Souto E.B., Zielinska A., Severino P., Batain F., Oliveira J., Alves T. (2021). Nanopesticides in agriculture: Benefits and challenge in agricultural productivity, toxicological risks to human health and environment. Toxics.

[15] Oftadeh M., Sendi J.J., Ebadollahi A., Setzer W.N., Krutmuang P. (2021). Mulberry protection through flowering-stage essential oil of *Artemisia annua* against the lesser mulberry pyralid *Glyphodes Pyloalis* Walker. Foods.

[16] Chandraboss V.L., Senthilvelan S., Natanapatham L.M., Murugavelu B., Loganathan B., Karthikeyan J. (2013). Photocatalytic effect of ag and ag/pt doped silicate non crystalline material on methyl violet—experimental and theoretical studies. J. Non. Cryst. Solids.

[17] Lee K.Y., Mooney D.J. (2012). Alginate: Properties and biomedical applications. Prog. Polym. Sci..

[18] Dey S., Sreenivasan K. (2014). Conjugation of curcumin onto alginate enhances aqueous solubility and stability of curcumin. Carbohydr. Polym..

[19] Ravichandran V., Jayakrishnan A. (2018). Synthesis and evaluation of anti-fungal activities of sodium alginate-amphotericin B conjugates. Int. J. Biol. Macromol..

[20] Campos E.V.R., de Oliveira J.L., Fraceto L.F., Singh B. (2015). Polysaccharides as safer release systems for agrochemicals. Agron. Sustain. Dev..

[21] Yihua Y., Mengdan Y., Jing X., Weiquan C., Ying Y., Guanghua H., Yue D., Ting Z., Mengqing J. (2020). A sodium alginate-based nano-pesticide delivery system for enhanced in vitro photostability and insecticidal efficacy of phloxine B. Carbohydr. Polym..

[22] Vinod V.T.P., Nazarzadeh Zare E., Makvandi P., Černík M. (2021). Nanoparticles and nanofibres based on tree gums: Biosynthesis and applications. Compr. Anal. Chem..

[23] Yang F.-L., Li X.-G., Zhu F., Lei C.-L. (2009). Structural Characterization of Nanoparticles Loaded with Garlic Essential Oil and Their Insecticidal Activity against *Tribolium castaneum* (Herbst) (Coleoptera: Tenebrionidae). J. Agric. Food Chem..

[24] Chen S., Yang K., Tuguntaev R.G., Mozhi A., Zhang J., Wang P.C., Liang X.J. (2016). Targeting tumor microenvironment with PEG-based amphiphilic nanoparticles to overcome chemoresistance. Nanomedicine.

[25] Suk J.S., Xu Q., Kim N., Hanes J., Ensign L.M. (2016). PEGylation as a strategy for improving nanoparticle-based drug and gene delivery. Adv. Drug Deliv. Rev..

[26] Lewicka K., Dobrzynski P., Rychter P. (2020). PLAGA-PEG-PLAGA terpolymer-based carriers of herbicides for potential application in environment-friendly, controlled release systems of agrochemicals. Materials.

[27] Shi X.B., Jiang L.L., Wang H.Y., Qiao K., Wang D., Wang K.Y. (2011). Toxicities and sublethal effects of seven neonicotinoid insecticides on survival, growth and reproduction of imidacloprid-resistant cotton aphid, *Aphis gossypii*. Pest Manage. Sci..

[28] Wang X., Yan M., Zhou J., Song W., Xiao Y., Cui C., Gao W., Ke F., Zhu J., Gu Z. (2021). Delivery of acetamiprid to tea leaves enabled by porous silica nanoparticles: Efficiency, distribution and metabolism of acetamiprid in tea plants. BMC Plant Biol..

[29] Fogel M.N., Schneider M.I., Desneux N., González B., Ronco A.E. (2013). Impact of the neonicotinoid acetamiprid on immature stages of the predator *Eriopis connexa* (Coleoptera: Coccinellidae). Ecotoxicology.

[30] Padmavathi P., Vasundhara N., Kovvuri S., Venugopal N. (2021). Synthesis and characterization of nano-acetamiprid—New plant safeguard nanomaterial. Am. J. Anal. Chem..

[31] Su Y., Ren X., Ma X., Wang D., Hu H., Song X., Cui J., Ma Y., Yao Y. (2022). Evaluation of the toxicity and sublethal effects of acetamiprid and dinotefuran on the predator *Chrysopa pallens* (Rambur) (Neuroptera: Chrysopidae). Toxics.

[32] Slattery M., Harper B., Harper S. (2019). Pesticide encapsulation at the nanoscale drives changes to the hydrophobic partitioning and toxicity of an active ingredient. Nanomaterials.

[33] Chavanpatil M.D., Khdair A., Patil Y., Handa H., Mao G., Panyam J. (2007). Polymer-surfactant nanoparticles for sustained release of water-soluble drugs. J. Pharm. Sci..

[34] Fakhri P., Amini B., Bagherzadeh R., Kashfi M., Latifi M., Yavari N., Asadi Kani S., Kong L. (2019). Flexible hybrid structure piezoelectric nanogenerator based on ZnO nanorod/PVDF nanofibers with improved output. RSC Adv..

[35] Bradford M.M. (1976). A rapid and sensitive method for the quantitation of microgram quantities of protein utilizing the principle of protein-dye binding. Anal. Biochem..

[36] Siegert K.J. (1987). Carbohydrate metabolism in *Manduca sexta* during late larval development. J. Insect Physiol..

[37] Fossati P., Prencipe L. (1982). Serum triglycerides determined colorimetrically with an enzyme that produces hydrogen peroxide. Clin. Chem..

[38] Van Asperen K. (1962). A study of housefly esterases by means of a sensitive colorimetric method. J. Insect Physiol..

[39] Oppenorth F.J., van der Pas L.J.T., Houx N.W.H. (1979). Glutathione S-transferase and hydrolytic activity in a tetrachlorvinphos-resistant strain of house fly and their influence on resistance. Pestic. Bioch. Physiol..

[40] Ebadollahi A., Sendi J.J., Setzer W.N., Changbunjong T. (2022). Encapsulation of *Eucalyptus largiflorens* essential oil by mesoporous silicates for effective control of the cowpea weevil, *Callosobruchus maculatus* (Fabricius) (Coleoptera: Chrysomelidae). Molecules.

[41] Kumar S., Chauhan N., Gopal M., Kumar R., Dilbaghi N. (2015). Development and evaluation of alginate–chitosan nanocapsules for controlled release of acetamiprid. Int. J. Biol. Macromol..

[42] Deng S., Gigliobianco M.R., Censi R., Di Martino P. (2020). Polymeric nanocapsules as nanotechnological alternative for drug delivery system: Current status, challenges and opportunities. Nanomaterials.

[43] Brown D., Ng’ambi J.W. (2017). Effect of polyethylene glycol 4000 supplementation on the performance of yearling male Pedi goats fed dietary mixture levels of Acacia karroo leaf meal and Setaria verticillata grass hay. Trop. Anim. Health Prod..

[44] Elshafie H.S., Camele I. (2021). Applications of absorbent polymers for sustainable plant protection and crop yield. Sustainability.

[45] Martău G.A., Mihai M., Vodnar D.C. (2019). The Use of Chitosan, Alginate, and Pectin in the Biomedical and Food Sector—Biocompatibility, Bioadhesiveness, and Biodegradability. Polymers.

[46] Adak T., Kumar J., Shakil N.A., Walia S. (2012). Development of Controlled release formulations of imidacloprid employing novel nano-ranged amphiphilic polymers. J. Environ. Sci. Health Part B.

[47] Patel S., Bajpai J., Saini R., Bajpai A.K., Acharya S. (2018). Sustained release of pesticide (Cypermethrin) from nanocarriers: An effective technique for environmental and crop protection. Process Saf. Environ. Prot..

[48] Bhan S., Mohan L., Srivastava C.N. (2014). Relative larvicidal potentiality of nano-encapsulated temephos and imidacloprid against *Culex quinquefasciatus*. J. Asia Pac. Entomol..

[49] Hemalatha N., Kumar K.R. (2021). Synthesis and characterization of PEG-cinnamon essential oil nanoparticles and their application as an insecticidal agent. J. Adv. Sci. Res..

[50] Keawchaoonm L., Yoksanm R. (2011). Preparation, characterization and in vitro release study of carvacrol-loaded chitosan nanoparticles. Colloids Surf. B..

[51] Takei T., Yoshida M., Hatate Y., Shiomori K., Kiyoyama S. (2008). Preparation of polylactide/poly(ε-caprolactone) microspheres enclosing acetamiprid and evaluation of release behavior. Polym. Bull..

[52] Bursell E., Downer R.G.H. (1981). The Role of Proline in Energy Metabolism. Energy Metabolism in Insects.

[53] Gall M.L., Behmer S.T. (2014). Effects of protein and carbohydrate on an insect herbivore: The vista from a fitness landscape. Integr. Comp. Biol..

[54] Klowden M.J. (2013). Physiological Systems in Insects.

[55] Arrese E.L., Soulages J.L. (2010). Insect fat body: Energy, metabolism, and regulation. Annu. Rev. Entomol..

[56] Ugrankar R., Theodoropoulos P., Akdemir F., Henne W.M., Graff J.M. (2018). Circulating glucose levels inversely correlate with Drosophila larval feeding through insulin signaling and SLC5A11. Commun. Boil..

[57] Wu M.Y., Ying Y.Y., Zhang S.S., Li X.G., Yan W.H., Yao Y.C., Shah S., Wu G., Yang F.L. (2020). Effects of diallyl trisulfide, an active substance from garlic essential oil, on energy metabolism in male moth *Sitotroga cerealella* (Olivier). Insects.

[58] Oftadeh M., Sendi J.J., Ebadollahi A. (2020). Toxicity and deleterious effects of *Artemisia annua* essential oil extracts on mulberry pyralid (*Glyphodes pyloalis*). Pestic. Biochem. Physiol..

[59] Nardini L., Christian R.N., Coetzer N., Ranson H., Coetzee M., Koekemoer L.L. (2012). Detoxification enzymes associated with insecticide resistance in laboratory strains of *Anopheles arabiensis* of different geographic origin. Parasit. Vectors.

[60] Hasheminia S.M., Sendi J.J., Jahromi K.T., Moharramipour S. (2011). The effects of *Artemisia annua* L. and *Achillea millefolium* L. crude leaf extracts on the toxicity, development, feeding efficiency and chemical activities of small cabbage *Pieris rapae* L. (Lepidoptera: Pieridae). Pestic. Biochem. Physiol..

[61] Ghormade V., Deshpande M.V., Paknikar K.M. (2011). Perspectives for nano-biotechnology enabled protection and nutrition of plants. Biotechnol. Adv..

